# Eye and Head Movements of Elite Baseball Players in Real Batting

**DOI:** 10.3389/fspor.2020.00003

**Published:** 2020-01-29

**Authors:** Yuki Kishita, Hiroshi Ueda, Makio Kashino

**Affiliations:** ^1^Department of Information and Communications Engineering, School of Engineering, Tokyo Institute of Technology, Tokyo, Japan; ^2^NTT Communication Science Laboratories, Nippon Telegraph and Telephone Co., Atsugi, Japan

**Keywords:** baseball batting, eye movements, hand-eye coordination, head movements, predictive saccades

## Abstract

In baseball, batters swing in response to a ball moving at high speed within a limited amount of time—about 0. 5 s. In order to make such movement possible, quick and accurate trajectory prediction followed by accurate swing motion with optimal body-eye coordination is considered essential, but the mechanisms involved are not clearly understood. The present study aims to clarify the strategies of eye and head movements adopted by elite baseball batters in actual game situations. In our experiment, six current professional baseball batters faced former professional baseball pitchers in a scenario close to a real game (i.e., without the batters informed about pitch type in advance). We measured eye movements with a wearable eye-tracker and head movements and bat trajectories with an optical motion capture system while the batters hit. In the eye movement measurements, contrary to previous studies, we found distinctive predictive saccades directed toward the predicted trajectory, of which the first saccades were initiated approximately 80–220 ms before impact for all participants. Predictive saccades were initiated significantly later when batters knew the types of pitch in advance compared to when they did not. We also found that the best three batters started predictive saccades significantly later and tended to have fewer gaze-ball errors than the other three batters. This result suggests that top batters spend slightly more time obtaining visual information by delaying the initiation of saccades. Furthermore, although all batters showed positive correlations between bat location and head direction at the time of impact, the better batters showed no correlation between bat location and gaze direction at that time. These results raise the possibility of differences in the coding process for the location of bat-ball contact; namely, that top batters might utilize head direction to encode impact locations.

## Introduction

**Figure 1:**
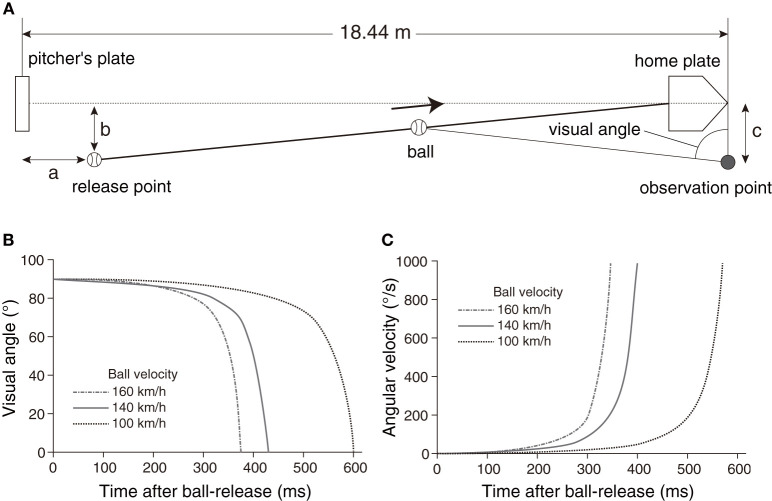
**(A)** Geometric relationship around a pitcher and a batter. The pitcher releases the ball at a release point toward the home plate, and the player looks at the ball from the observation point. The parameters a = 1.8 m, b = 1.0 m, and c = 1.0 m were used for the simulation on ball trajectories. **(B)** Simulation results of visual angle of the ball from the observation point with different ball velocities (100, 140, 160 km/h). **(C)** Simulation results of angular velocities of the ball from the observation point with different ball velocities (100, 140, 160 km/h).

When we intercept or hit a moving object, we have to estimate its trajectory and initiate action corresponding to the appropriate position and timing. Baseball batting is one of the most difficult interception tasks that humans can perform. In baseball, the pitcher throws a ball toward the home plate, which is about 18 m away, and the batter hits the ball passing through the home plate with a bat (Figure 1A). When the pitcher throws the ball at 160 km/h, the time from ball-release to bat-ball contact is <400 ms (Figure 1B). The angular velocity of the ball exceeds 800 deg/s (Figure 1C), so it is presumably impossible to track the ball during its entire flight. Within that period, the batter has to estimate the ball trajectory and generate swing motion with accurate spatial and temporal bat controls. Moreover, since the batter is also required to adjust to many types of pitch (fastball, breaking ball, changeup, etc.), it is more difficult to predict the trajectory of the ball in an actual game. As a result, even most professional league batters do not reach the batting average of 30%. However, some batters (though only a limited number) leave a batting average of 30% or more every year. Here, focusing on the visual strategy of professional baseball players, we attempted to figure out whether there are differences in the strategy between highly elite players and other elite players.

Gaze movement strategies are known to vary depending on the type of hitting sport. In baseball, Fogt and Zimmerman (2014) measured eye movement and head rotation when a baseball batter sees a thrown ball (without a swing) and found that batters track the ball mainly using head rotation rather than eye movement. A recent study by Higuchi et al. (2018) showed similar results, even when batters actually hit the ball thrown by a pitching machine. In contrast, in cricket, where batters hit the ball after it has bounced on the ground, the batters continuously track the ball in the fovea for a while after the ball is released and then make a predictive saccade toward the area where the ball will bounce (Land and McLeod, 2000; Croft et al., 2010; Mann et al., 2013). Predictive saccades have also been observed in other hitting sports where ball bounces occur, such as tennis (Williams et al., 1998), table tennis (Ripoll et al., 1986; Land and Furneaux, 1997), and squash (Hayhoe et al., 2012). Thus, the role of the predicted saccade is considered to facilitate tracking of the ball after bouncing by looking at the predicted ball-bounce location, which has more information directly related to performance.

Previous studies have shown that gaze movement strategies are related to hitting and interception performance. Land and McLeod (2000) revealed that cricket batters utilize a predictive saccade toward the future ball-bounce position, and better batters initiate a predictive saccade earlier and track the ball more precisely. Mann et al. (2013) reported that elite cricket batters show an exquisite head-ball coupling movement that keeps the ball direction from the head constant. Bahill and LaRitz (1984) measured eye and head movements when baseball batters faced a physically simulated ball without swinging and showed that the batter followed the ball using both smooth pursuit eye movements and head rotation, where professional batters had better tracking ability than amateur batters. Furthermore, in laboratory studies, the accuracy of smooth pursuit eye movement has been shown to be positively correlated with perceptual localization (Spering et al., 2011) and manual interception of moving targets (Fooken et al., 2016).

Although many studies have been done on gaze movement strategies in hitting sports, there has been little focus on elite batters in real batting situations, especially in baseball. Elite batters may present an ideal strategy to underpin performance in a strict environment close to the actual game. Moreover, the factors behind what it takes to be an outstanding batter (vs. a good batter) remain unclear. Considering these points, we measured the eye and head movements of two groups of professional baseball batters (top league and farm league), including a scenario close to the actual game; batters are not announced the pitch type in advance. We expected that the presence or absence of prior information about pitch types may affect gaze movement strategies, considering the finding that eye movements are influenced by the predictability of the target trajectory (Findlay, 1981; Kowler et al., 1984; Becker and Fuchs, 1985; Shelhamer and Joiner, 2003). The presence or absence of prior pitch information could reveal differences in skill levels that would not be apparent in a simple predictable ball trajectory.

## Methods

### Participants

Six professional baseball field players from Nippon Professional Baseball (NPB), the top baseball league in Japan, took part in the off-season. Three participants belonged to top teams (Top 1–3: *M* = 26.7 years) and the other three to farm teams (Farm 1–3: *M* = 24.7 years) in the most recent season. Only Farm 2 was a right-handed batter, and the others were all left-handed. The level difference between the top group and the farm group was distinctive. The top group players had more than 40 at-bats in the top league games, and the farm group players never played as batters in the top league games in the previous season. NPB players can be considered highly elite, even if they belong to the farm league. Baseball is one of the most popular sports in Japan, which ranks top in the World Baseball Softball Confederation ranking in 2019. The number of NPB players, including the top league and farm league, is only about 800. All the participants provided written informed consent prior to the experiments. This study was approved by the Ethics and Safety Committees of NTT Communication Science Laboratories and were in accordance with the Declaration of Helsinki.

### Apparatus

**Figure 2:**
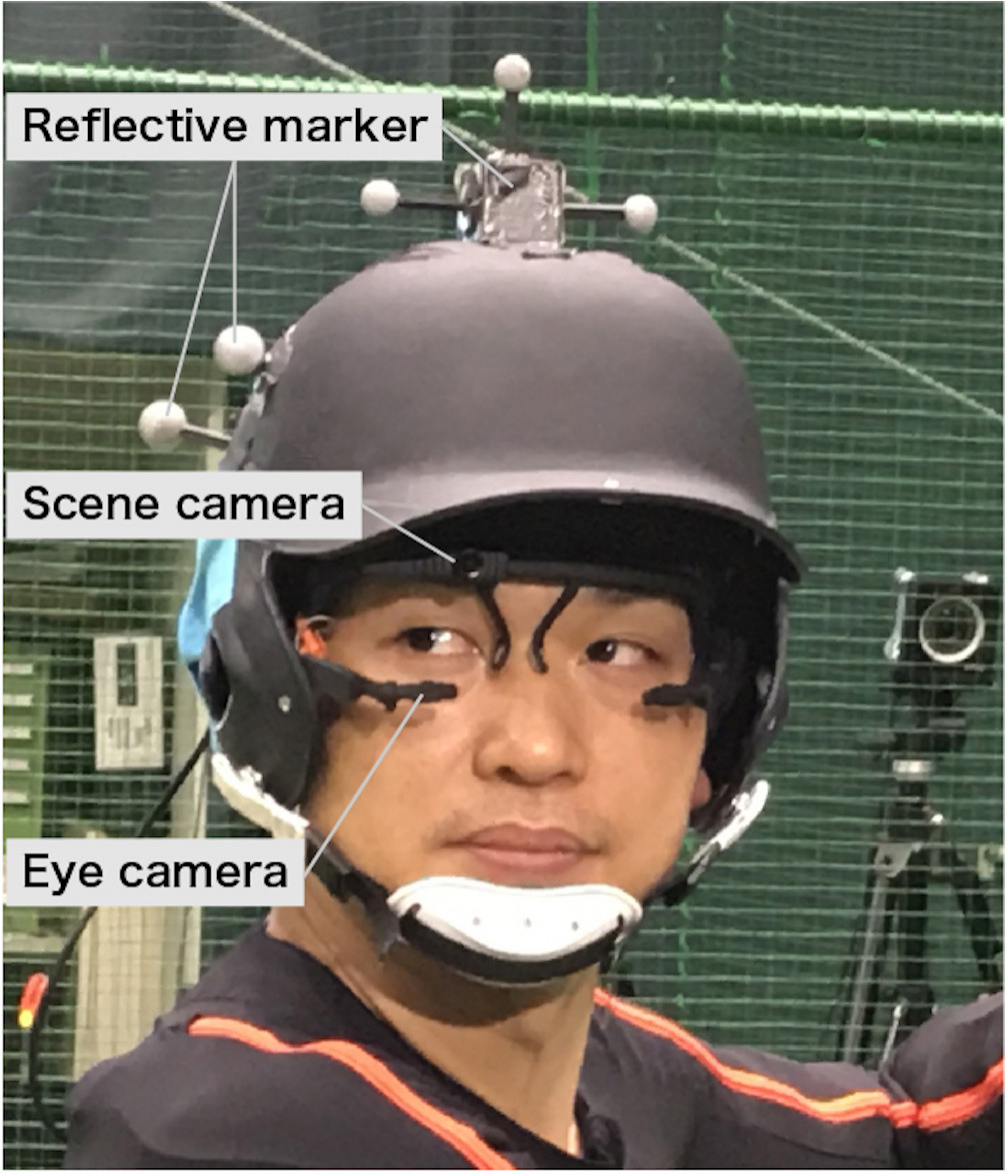
Participant wearing experimental equipment. The eye-tracker is equipped with eye cameras (for recording images of eyes) and a scene camera (for recording participants' view). The helmet is attached with reflective markers for motion capture. The helmet is tightly fixed to the participant's head with chin strap and headband. Written informed consent was obtained from the individual for the publication of this image.

The experimental setup is shown in Figure 2. Participants wore a batting helmet and a wearable eye-tracker (Pupil headset, Pupil Labs GmbH, Germany). The eye-tracker recorded the eye movements of the right eye with an eye camera at a sampling rate of 200 Hz and the video from the participant's point of view with a scene camera at a sampling rate of 120 Hz. During the experiment, we monitored gaze locations and the image of the eye camera in real-time, and recalibrated the eye-tracker whenever the camera slipped. The movements of the participant's head and the bat were measured by recording the position of reflective markers attached to the helmet and the top of the bat, respectively, at a sampling frequency of 240 Hz with an optical motion capture system (Optitrack, NaturalPoint, Inc., United States). The helmet was fixed tightly to the head with a headband and chin strap to prevent contact between the eye-tracker and helmet and to achieve accurate measurement of head motions during swing motions. Events such as ball releases and bat-ball contacts were recorded using a high-speed camera at a sampling rate of 300 Hz. Time synchronization among these devices was achieved by lighting LEDs simultaneously at the time of the initiation of the motion capture system. Pitch speed was measured by a 3D Doppler radar system (TrackMan Baseball, TrackMan, Inc., Denmark).

### Procedure and Design

Former professional pitchers threw against participants. One participant (Farm 1) faced Pitcher 1 (fastball: *M* = 104.9 km/h, *SD* = 1.40 km/h; curveball: *M* = 95.2 km/h, *SD* = 1.42 km/h), and the other participants faced Pitcher 2 (fastball: *M* = 128.8 km/h, *SD* = 1.38 km/h; curveball: *M* = 104.9 km/h, *SD* = 1.40 km/h). Prior to experimentation, the eye-tracker was calibrated using “manual marker calibration” provided in the eye-tracker's operating software. Specifically, participants were instructed to stand in the batter box and look at a calibration target placed 1.5–2 m in front of them. The researcher changed the target positions so as to cover all ball trajectories. The number of calibration points was set to more than 10. This calibration procedure was performed every time the eye-tracker slipped. In the experiment, the pitcher threw either a fastball or a curveball under the *known condition*, in which the pitch type was told to the participant in advance, and the *unknown condition*, in which the pitch type was not told to the batter in advance. Thus, the unknown conditions are closer to an actual game situation, reducing the predictability of the ball trajectory and making the task more difficult. Since we tested only fastball in the known condition, there were three experimental conditions in total: *known fastball, unknown fastball*, and *unknown curveball*. These three conditions were held in random order. Participants were instructed to hit all strike balls. The experiment was repeated until there were 11 bat-ball contacts for each of the three conditions.

### Data Analysis

**Figure 3:**
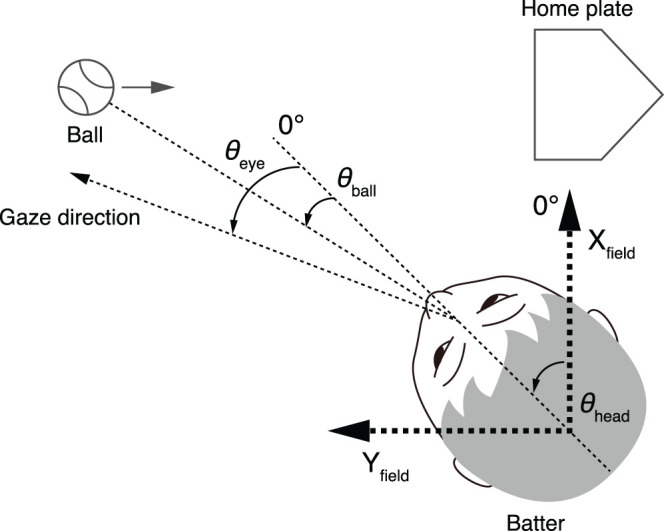
Geometry of parameters. Directions of Y_field_ axes are parallel to the pitcher-home plate directions, and X_field_ axis is vertical to Y_field_ axis. The origin of the X_field_-Y_field_ coordinate was set in the center of the head. *θ*_eye_, eye position; *θ*_ball_, ball direction relative to head center; *θ*_head_, head direction (angle subtended by head direction and X_field_ axis).

The coordinate system was defined as shown in Figure 3, similar to Higuchi et al. (2018). The direction of the Y_field_ axis was set parallel to the pitcher-home plate direction, and that of the X_field_ axis was orthogonal to the Y_field_ axis. The origin of the X_field_-Y_field_ coordinate was set in the center of the head, so it moved with the participant's translational movements. The effect of the head translation on gaze shift was not evaluated because its contribution was relatively small compared to the head rotation. The effect of the head translation on the ball direction (*θ*_ball_) is taken into account because it was calculated on the basis of scene camera images mounted on the participant's head. The data of the left-handed batters were inverted to fit this coordinate system.

The head direction (*θ*_head_) indicates the angle between the head direction and the X_field_ axis. Three out of eight motion markers attached on the helmet formed a transverse plane, and the head direction was calculated on the basis of the positions of the three markers projected on the ground. The motion capture data were resampled to match the sampling frequency of the eye position data. The ball (*θ*_ball_) and gaze (*θ*_eye_) directions were defined relative to the head direction (*θ*_head_). The ball positions were manually digitized from the footage of the scene camera and interpolated with third-order spline interpolation so as to match the sampling frequency of the gaze position data. The gaze position data were exported as x-y coordinates in the scene camera image and smoothed with a second-order Savitzky-Golay filter with a window size of nine points. Finally, the ball and gaze position expressed in the scene camera coordinates were converted into angular parameters relative to the head while taking into account the head tilt, obtained from motion capture data, and correcting the distortion caused by the wide lens. Therefore, the ball and gaze direction in the field coordinate systems can be expressed as (*θ*_head_ + *θ*_ball_) and (*θ*_head_ + *θ*_eye_), respectively. Please note that *θ*_head_ + *θ*_ball_ is independent of the participant's head direction.

Eye movements exceeding the maximum velocity of 150 deg/s and the maximum acceleration of 6,000 deg/s^2^ were defined as saccades; we adopted conservative criteria to detect only distinct saccades. Saccade detection was further verified by visual inspection. The onset of a saccade was defined as the time at which the acceleration of the saccade reached its maximum value. Only data from trials where a bat-ball contact occurred were subject to further analyses (to align data at the time of the bat-ball contact). Trials that yielded no correct data were also removed. For each participant and condition, the number of trials used in the analysis was at least eight out of 11 trials in which a bat-ball contact occurred.

### Statistical Analyses

For each participant and each condition, mean values were calculated for the cumulative error between the gaze and the ball positions during the 300 ms before the bat-ball contact, the time of the first saccade, and the contribution of the eye movements to the total amount of gaze shift from ball release to bat-ball contact. For these three variables, to examine the effects of the participant level and pitch conditions, we performed a 2 (top league batters and farm league batters) × 3 (known fastball, unknown fastball, and unknown curveball) mixed analysis of variance (ANOVA). Bonferroni-corrected *post hoc t*-tests with the alpha level of 0.05 were used for multiple comparisons.

To investigate the special relationships between visual strategies and swing motions, we conducted correlation analyses calculating Pearson's correlation coefficients between the bat-top positions and the three variables related to visual strategies (eye position *θ*_eye_, head direction *θ*_head_, gaze direction *θ*_head_ + *θ*_eye_) at the time of bat-ball contact. In order to increase the number of samples, data of all three pitch conditions have been combined.

## Results

**Figure 4:**
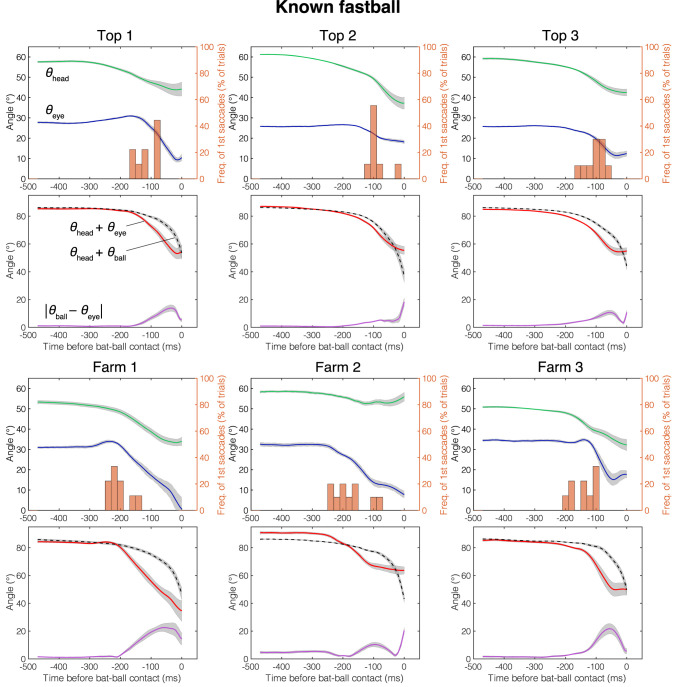
Mean eye position, gaze direction, and head direction for each player in known fastball condition. Green line, head direction (*θ*_head_); Blue line, eye position (*θ*_eye_); Red line, gaze direction (*θ*_head_ + *θ*_eye_); Dotted line, ball direction in field coordinate (*θ*_head_ + *θ*_ball_); Purple line, Horizontal error between gaze and ball (|*θ*_eye_ − *θ*_ball_|); Histogram, percentage that first saccade occurs; Gray shaded area, standard error. Time is aligned with the time at bat-ball contact.

**Figure 5:**
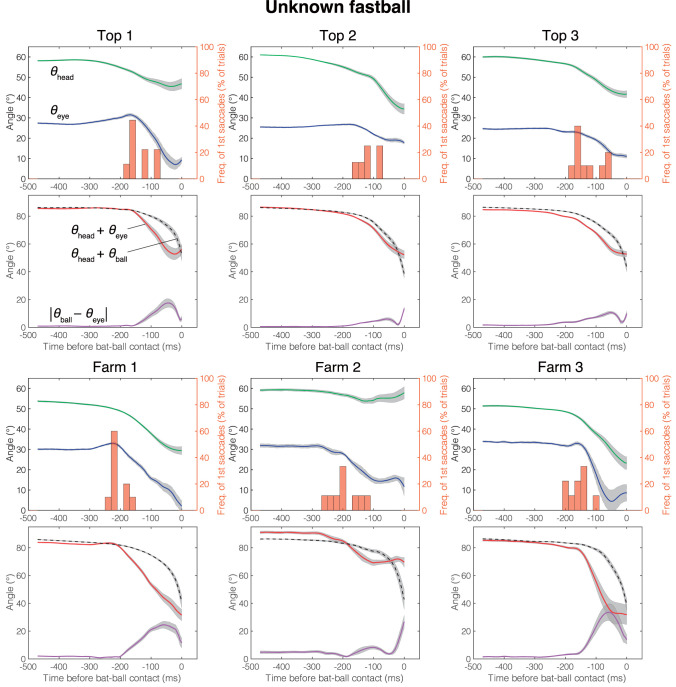
Mean eye position, gaze direction, and head direction for each player in unknown fastball condition. Green line, head direction (*θ*_head_); Blue line, eye position (*θ*_eye_); Red line, gaze direction (*θ*_head_ + *θ*_eye_); Dotted line, ball direction in field coordinate (*θ*_head_ + *θ*_ball_); Purple line, Horizontal error between gaze and ball (|*θ*_eye_ − *θ*_ball_|); Histogram, percentage that first saccade occurs; Gray shaded area, standard error. Time is aligned with the time at bat-ball contact.

**Figure 6:**
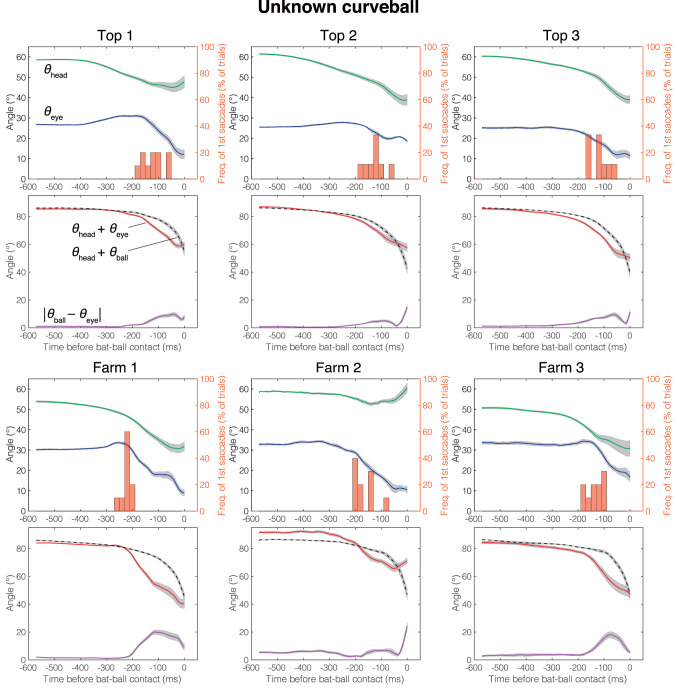
Mean eye position, gaze direction, and head direction for each player in unknown curveball condition. Green line, head direction (*θ*_head_); Blue line, eye position (*θ*_eye_); Red line, gaze direction (*θ*_head_ + *θ*_eye_); Dotted line, ball direction in field coordinate (*θ*_head_ + *θ*_ball_); Purple line, Horizontal error between gaze and ball (|*θ*_eye_ − *θ*_ball_|); Histogram, percentage that first saccade occurs; Gray shaded area, standard error. Time is aligned with the time at bat-ball contact.

The mean head direction and the gaze position of the known fastball, unknown fastball, and unknown curveball trials aligned at the time of the bat-ball contact are shown in Figures 4–6, respectively. General characteristics seen in all participants and conditions are as follows. Participants tracked the ball smoothly for a while after the ball release and at some point, started a saccade, and a quick head movement to shift gaze position to the future ball position (predictive saccades and head rotations). Participants mainly used their head movements to track the ball until they began saccadic eye movements. Participants showed predictive saccades in almost every trial. Top 1 showed a typical vestibulo-ocular reflex, where the head is rotated toward the flight direction of the ball, and the eyes are moved in the opposite direction while stabilizing the gaze at the ball position (about −400 to −200 ms in Figures 4–6). Farm 2 had a gap of about 5 degrees between ball and gaze position between 480 and 250 ms before the bat-ball contact, suggesting that the ball was being tracked without foveation.

**Figure 7:**
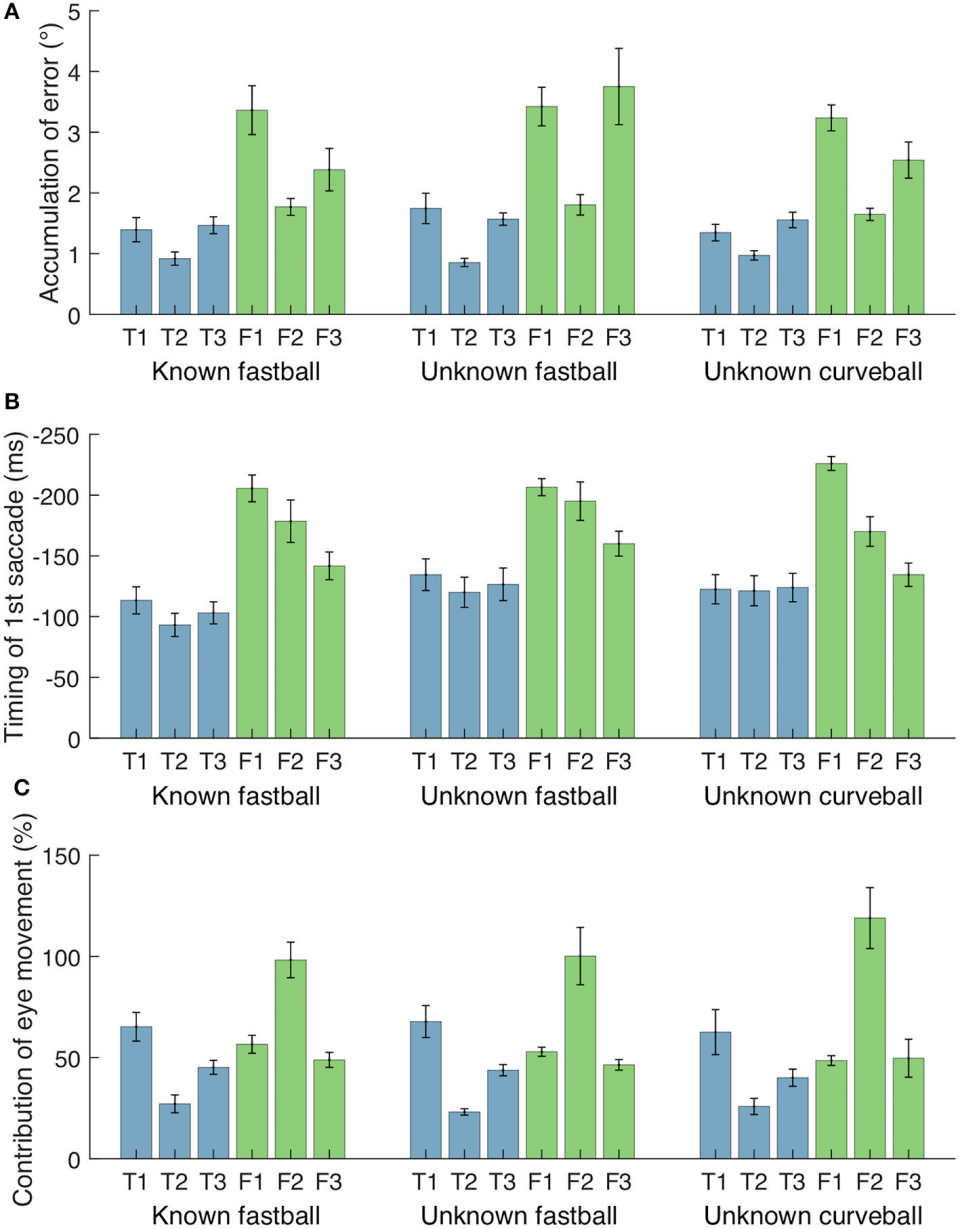
Accumulative error between gaze and ball, timing of first saccade, and contribution of eye movements for each participant and condition. **(A)** Accumulation of gaze-ball error during 300 ms before bat-ball contacts. **(B)** Initiation time of first saccades from bat-ball contacts. **(C)** Contribution of eye movements (effect of change in *θ*_eye_) to the change in gaze direction (*θ*_head_ + *θ*_eye_) from ball release to bat-ball contact. Error bars represent standard error.

Figure 7A shows the cumulative error between the gaze and the ball positions (|*θ*_ball_ − *θ*_eye_|) during 300 ms before the bat-ball contact for each participant. To evaluate the effects of the batter level (top batters vs. farm batters) and pitch conditions (difference in the predictability of ball trajectory) on tracking performance, a two-way mixed ANOVA was conducted on the cumulative error. The result showed a marginally significant difference in that the top league batters had better tracking performance than the farm league batters [*F*_(1, 4)_ = 6.77, *p* = 0.06, $\eta_{G}^{2}$ = 0.59]. On the other hand, no main of the pitch conditions [*F*_(2, 8)_ = 2.09, *p* = 0.19, $\eta_{G}^{2}$ = 0.06] or interaction was found [*F*_(2, 8)_ = 0.85, *p* = 0.46, $\eta_{G}^{2}$ = 0.03].

The mean initiation time of the first saccade in each trial is shown in Figure 7B. For all participants and pitch conditions, the first saccades were started about 80–220 ms before the bat-ball contact time. A two-way mixed ANOVA on initiation times of the first saccade with the groups and conditions as factors revealed that the farm batters made their initial saccade earlier than the top batters [*F*_(1, 4)_ = 9.92, *p* = 0.03, $\eta_{G}^{2}$ = 0.69]. Moreover, a significant main effect was found for different conditions [*F*_(2, 8)_ = 4.96, *p* = 0.04, $\eta_{G}^{2}$ = 0.11]. *Post-hoc* tests showed that the first saccades were initiated significantly earlier in the unknown fastball condition than in the known one [*t*_(4)_ = 6.20, *p* < 0.01]. The differences between the other pairs were not significant [known fastball vs. unknown curveball: *t*_(4)_ = 1.92, *p* = 0.13; unknown fastball vs. unknown curveball: *t*_(4)_ = 0.96, *p* = 0.39]. No interaction effect was found [*F*_(2, 8)_ = 1.26, *p* = 0.33, $\eta_{G}^{2}$ = 0.03].

The contribution of eye movements (i.e., the effect of change in *θ*_eye_) to the change in gaze direction (*θ*_head_ + *θ*_eye_) from ball release to bat-ball contact was also examined (Figure 7C). The shifts of these angular variables were calculated by subtracting the values at bat-ball contact from those at ball release. The contribution of eye movement varies widely from about 25% to over 100% among participants. The value above 100% (as seen in Farm 2) means that, at the time of bat-ball contact, the head was rotated in the opposite direction to the ball flight (see the green line of Farm 2 in Figures 4–6). No main effect of the group condition [*F*_(1, 4)_ = 1.25, *p* = 0.33, $\eta_{G}^{2}$ = 0.23] or the pitch condition [*F*_(2, 8)_ = 0.17, *p* = 0.85, $\eta_{G}^{2}$ < 0.01] was observed. The interaction effect was also not found [*F*_(2, 8)_ = 0.90, *p* = 0.44, $\eta_{G}^{2}$ < 0.01].

**Figure 8:**
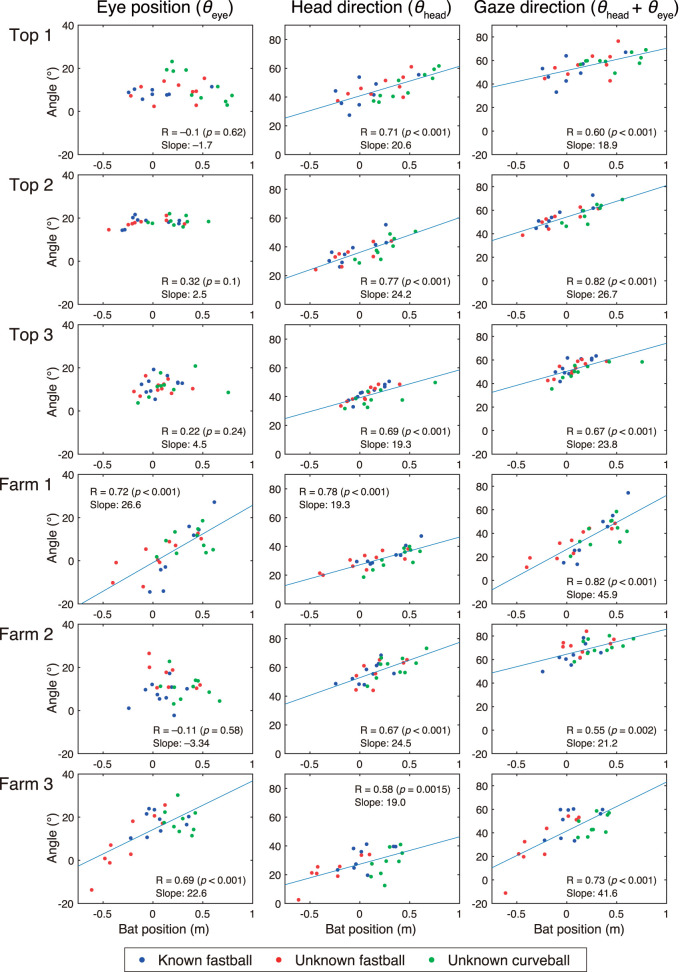
Correlation between bat positions and eye positions, head directions, and gaze directions at time of bat-ball contact. Horizontal axis shows bat top position at bat-ball contact. Zero means the bottom position of the home plate, a positive value means the pitcher direction, and a negative value means the catcher direction. Vertical axis varies depending on the column. Eye position (*θ*_eye_), head direction (*θ*_head_), and gaze direction (*θ*_head_ + *θ*_eye_) are shown from the left column. Plot colors correspond to the experimental conditions: blue for known fastball, red for unknown fastball, and green for unknown curveball. Regression lines are shown when correlations are statistically significant.

The results of correlation analyses for the special relationships between the bat-top positions and the three variables related to visual strategies (eye position *θ*_eye_, head direction *θ*_head_, gaze direction *θ*_head_ + *θ*_eye_) at the time of bat-ball contact are shown in Figure 8. For the bat position (x-axis), zero means the bottom position of the home plate, a positive value means the pitcher direction, and a negative value means the catcher direction. All participants showed a significant correlation between bat positions and gaze direction (*R* = 0.55–0.82) and bat positions and head directions (*R* = 0.58–0.80). However, only the Farm batters (Farm 1 and Farm 2) showed a significant correlation between bat positions and eye directions (*R* = 0.77 and *R* = 0.68, respectively).

## Discussion

In this study, we examined the eye and head movements of six professional baseball batters in a real batting task. We found that the batters estimated the ball trajectory by a combination of eye movements and head rotations while constantly utilizing predictive saccades to future ball locations. These results were consistent regardless of the batters' level (top/farm) or the pitch conditions (known fastball/unknown fastball/unknown curveball). For a certain period after the ball release, most batters followed the ball using head rotation rather than eye movements. These results are consistent with behaviors observed in top cricket (Mann et al., 2013) and baseball (Fogt and Zimmerman, 2014) batters. About 50–250 ms before the bat-ball contact, all participants made a predictive saccade to around the future ball trajectory in almost every trial. Bahill and LaRitz (1984) also showed that baseball batters sometimes make a predictive saccade when they face a physically simulated ball. However, here, we found that elite baseball batters utilize a predictive saccade almost every time in real batting situations.

The differences due to the skill level of the batters were confirmed in the gaze movement strategies. Top league batters initiated predictive saccades significantly later than farm league batters. Moreover, the top league batters tended to have a smaller error between gaze and ball than farm league batters. Since the gaze-ball error was largest during the saccade period (Figures 4–6), the top league batters, intentionally or not, would utilize a strategy to foveate the ball longer by delaying the generation of a predictive saccade. Previous studies on behavioral differences due to the batter's skill level have mostly compared groups with a huge gap in skill (e.g., professional vs. amateur). Thus, this result is surprising in that observable differences related to skill level were found even among highly elite batters. Land and McLeod (2000) reported that professional cricket batters initiate a predictive saccade to ball bounce locations earlier than amateur batters, unlike our results. In cricket, visual information of the ball before the bounce would be less important than that after the bounce, so batters might start predictive saccades earlier, preparing for tracking the ball after the bounce. The advantages of better tracking ability have also been demonstrated in laboratory experiments (e.g., Spering and Montagnini, 2011; Fooken et al., 2016; Spering and Schütz, 2016). Even so, similar results in the real field and with elite batters raise the possibility that these characteristics (better tracking abilities yielded by late predictive saccades) form the basis of what it takes to be a top batter.

Keeping an eye on the ball longer leads to a more accurate estimation of the ball trajectory. However, the period of visual information during ball flight available for swing movement is constrained by the latency of the visuomotor response. Simple reaction time to a light flash, for example, typically take 200 to 250 ms (Meyer et al., 1988). In contrast, in the case of adjustment of a reaching movement (in response to a sudden target shift or jump), the reaction times will be much shorter (up to 110 ms; Brenner and Smeets, 1997). Interestingly, in this study, the top league batters started saccades about 120 ms before the bat-ball contact. This timing is very close to the reaction time seen in the online adjustment response. In baseball batting, it is easy to imagine that online bat control plays an important role in light of the fact that batters sometimes stop the swing after starting it—although how far batters can change the swing motion withstanding the inertia of an accelerated bat remains a critical question. In addition, considering that new visual information cannot be obtained during a saccade due to saccade suppression, the top batters, who obtain visual information by keeping their eye on the ball up to the time limit that can be reflected in action, may have adopted the optimal eye movement strategy. The farm batters, on the other hand, made the predictive saccade (or shift the gaze away from the ball) earlier than the top batters. This means that the farm batters might miss available visual information. High-level pitchers often throw a ball whose trajectory looks a fastball until it starts curving just in front of a batter (e.g., moving fastball). For example, if a batter can track a ball 100 ms longer (a ball travels about 3–4 m in this period), the adjustability for such kind of pitches will increase.

Saccade timing in the unknown fastball condition was significantly earlier than that in the known fastball condition. The predictability of ball trajectories in the unknown conditions is assumed to be lower than that in the known condition. Many previous studies have suggested that the target can be tracked more easily when its trajectory is more predictable (Findlay, 1981; Kowler et al., 1984; Becker and Fuchs, 1985; Shelhamer and Joiner, 2003). Although we cannot provide a clear answer as to why the time of predictive saccades is earlier in the unknown condition, our results demonstrate that the time of predictive saccades is affected by prior knowledge of target trajectory. Furthermore, no significant difference was observed in the timing of the predictive saccades between unknown fastball and unknown curveball, even with a large difference in ball speed (about 15–25 km/h). These results suggest that the predictive saccades are not driven by external factors (e.g., ball position, velocity) but rather by some internal factors such as prior information of ball trajectories and swing motion.

All batters showed a correlation between bat positions and head directions at the time of bat-ball contact. On the other hand, while two of the three farm batters showed a correlation between bat position and eye position, none of the top batters showed the same correlation. This suggests that the top batters may encode the location of a bat-ball contact on the basis of their head direction, while the farm batters encode it on the basis of the combination of head direction and eye position. When reaching a target, we need to calculate the limb-centered target location by combining target locations expressed by eye-centered, head-centered, and torso-centered coordination (Land and Tatler, 2009). The top league batters may skip part of this process by keeping their eye position relative to the head constant. This shortcut might shorten the visuo-motor delay, and therefore, top league batters foveate the ball longer by delaying the initiation of the predictive saccades. Most participants continued to rotate their head until the bat-ball contact, so it is plausible that they made head rotation according to the prediction of ball trajectories. Neurons encoding head directions (head-direction cell) have been found in several brain regions of various mammals (Taube et al., 1990; Robertson et al., 1999; Yoder and Taube, 2009), some of which have been reported to encode future head directions (Blair and Sharp, 1995). One possibility is, therefore, that the head-direction cell signals of future head directions are used for adjustment of swing motions.

While all participants always adopted predictive saccades, their functional significance is not clear. A straightforward interpretation would be that by moving the gaze position in advance, more detailed visual information on a bat and/or a ball is obtained. Bahill and LaRitz (1984) offered the same interpretation. However, as mentioned earlier, considering the delay in the visuo-motor process, it seems impossible to reflect the very last visual information in the swing motion. The time between the ball passing through gaze location and the bat-ball contact is <50 ms, which is much less than the possible reaction time in online adjustment responses. However, learning of the ball and/or swing trajectories from the visual information obtained after predictive saccades would be useful for future plays. Another possibility is to make the representation of an impact position by locating their gaze in advance. During a reaching movement, the gaze reaches a target location before the hand in order to represent a target location in the eye-centered coordinate (Crawford et al., 2004). Similar mechanisms might work in baseball batting. A saccadic movement to the predicted impact point could pre-encode the position information in the brain with the eye-centered coordinate.

In this study, we measured the eye and body movements of professional baseball batters during baseball batting in a situation similar to actual games, and we found that even among highly skilled batters, there were differences in their gaze shifting and hand-eye coordination strategies. In addition, we revealed that this subtle but important difference in skill level was primarily related to the timing of the predictive saccade detected in almost all trials, unlike the previous studies (Fogt and Zimmerman, 2014; Higuchi et al., 2018). These previous studies have shown that batters mainly use head rotations but our results show that the contributions of eye movements to the total gaze sift varied from about 20% to more than 100% depending on batters (Figure 7C). However, it is not clear from the results of the present experiment whether the reason for the discrepancy with the previous studies is due to the difference in participants (e.g., amateur vs. professional) or experimental conditions (e.g., pitching machines vs. human pitchers). Thus, it is necessary to further study the gaze shift strategy with a broader range of participants and various experimental settings. In addition, the functional significance of the predictive saccades also remains a matter of speculation. In this regard, we expect that further understanding will be gained by reproducing extreme conditions such as those found in sports in laboratory experiments where factor control is relatively simple. Furthermore, because the number of participants in this type of study is often limited, we cannot rule out the possibility that this study might not capture all the characteristics of elite baseball batters. Thus, further studies are also needed in this respect. In any case, this is the first study assessing the body-eye coordination among highly skilled batters, and measuring the behaviors of highly skilled batters in extreme conditions such as this study is critical to understand the optimal strategy of body-eye coordination. Above all, these insights can help improve the skills of top batters.

## Data Availability Statement

The datasets generated for this study are available on request to the corresponding author.

## Ethics Statement

The studies involving human participants were reviewed and approved by NTT Communication Science Laboratories Research Ethics Committee. The patients/participants provided their written informed consent to participate in this study. Written informed consent was obtained from the individual(s) for the publication of any potentially identifiable images or data included in this article.

## Author Contributions

YK, HU, and MK conceived and designed the experiments and interpreted data. YK and HU performed the experiment. YK conducted data analysis and drafted the manuscript. HU and MK edited and revised the manuscript, and approved the final version.

### Conflict of Interest

YK and MK belong to the Tokyo Institute of Technology, and MK and HU were employees of NTT Communication Science Laboratories, which is a basic-science research section of Nippon Telegraph and Telecommunication Corporation (NTT). This does not alter the authors' adherence to policies of Frontiers.

## References

[B1] Bahill A. T. LaRitz T. (1984). Why can't batters keep their eyes on the ball? Am. Sci. 72, 249–253.

[B2] Becker W. Fuchs A. F. (1985). Prediction in the oculomotor system: smooth pursuit during transient disappearance of a visual target. Exp. Brain Res. 57, 562–575. 10.1007/BF00237843 3979498

[B3] Blair H. Sharp P. (1995). Anticipatory head direction signals in anterior thalamus: evidence for a thalamocortical circuit that integrates angular head motion to compute head direction. J. Neurosci. 15, 6260–6270. 10.1523/JNEUROSCI.15-09-06260.1995 7666208 PMC6577663

[B4] Brenner E. Smeets J. B. J. (1997). Fast responses of the human hand to changes in target position. J. Mot. Behav. 29, 297–310. 10.1080/00222899709600017 12453772

[B5] Crawford J. D. Medendorp W. P. Marotta J. J. (2004). Spatial transformations for eye–hand coordination. J. Neurophysiol. 92, 10–19. 10.1152/jn.00117.2004 15212434

[B6] Croft J. L. Button C. Dicks M. (2010). Visual strategies of sub-elite cricket batsmen in response to different ball velocities. Hum. Mov. Sci. 29, 751–763. 10.1016/j.humov.2009.10.004 20031242

[B7] Findlay J. M. (1981). Spatial and temporal factors in the predictive generation of saccadic eye movements. Vision Res. 21, 347–354. 10.1016/0042-6989(81)90162-0 7269312

[B8] Fogt N. F. Zimmerman A. B. (2014). A method to monitor eye and head tracking movements in college baseball players. Optom. Vis. Sci. 91, 200–211. 10.1097/OPX.0000000000000148 24394952

[B9] Fooken J. Yeo S. H. Pai D. K. Spering M. (2016). Eye movement accuracy determines natural interception strategies. J. Vis. 16:1. 10.1167/16.14.1 27802509

[B10] Hayhoe M. M. McKinney T. Chajka K. Pelz J. B. (2012). Predictive eye movements in natural vision. Exp. Brain Res. 217, 125–136. 10.1007/s00221-011-2979-2 22183755 PMC3328199

[B11] Higuchi T. Nagami T. Nakata H. Kanosue K. (2018). Head-eye movement of collegiate baseball batters during fastball hitting. PLoS ONE 13:e0200443. 10.1371/journal.pone.0200443 30016367 PMC6049917

[B12] Kowler E. Martins A. J. Pavel M. (1984). The effect of expectations on slow oculomotor control—IV. Anticipatory smooth eye movements depend on prior target motions. Vision Res. 24, 197–210. 10.1016/0042-6989(84)90122-6 6719834

[B13] Land M. Tatler B. (2009). Looking and Acting: Vision and Eye Movements in Natural Behaviour. Oxford: Oxford University Press.

[B14] Land M. F. Furneaux S. (1997). The knowledge base of the oculomotor system. Philos. Trans. R. Soc. London. Ser. B Biol. Sci. 352, 1231–1239. 10.1098/rstb.1997.0105 9304689 PMC1692006

[B15] Land M. F. McLeod P. (2000). From eye movements to actions: how batsmen hit the ball. Nat. Neurosci. 3, 1340–1345. 10.1038/81887 11100157

[B16] Mann D. L. Spratford W. Abernethy B. (2013). The head tracks and gaze predicts: how the world's best batters hit a ball. PLoS ONE 8:e58289. 10.1371/journal.pone.0058289 23516460 PMC3596397

[B17] Meyer D. E. Osman A. M. Irwin D. E. Yantis S. (1988). Modern mental chronometry. Biol. Psychol. 26, 3–67. 10.1016/0301-0511(88)90013-0 3061480

[B18] Ripoll H. Bard C. Paillard J. (1986). Stabilization of head and eyes on target as a factor in successful basketball shooting. Hum. Mov. Sci. 5, 47–58. 10.1016/0167-9457(86)90005-9

[B19] Robertson R. G. Rolls E. T. Georges-François P. Panzeri S. (1999). Head direction cells in the primate pre-subiculum. Hippocampus 9, 206–219. 10.1002/(SICI)1098-1063(1999)9:3<206::AID-HIPO2>3.0.CO;2-H 10401637

[B20] Shelhamer M. Joiner W. M. (2003). Saccades exhibit abrupt transition between reactive and predictive, predictive saccade sequences have long-term correlations. J. Neurophysiol. 90, 2763–2769. 10.1152/jn.00478.2003 14534279

[B21] Spering M. Montagnini A. (2011). Do we track what we see? Common versus independent processing for motion perception and smooth pursuit eye movements: A review. Vision Res. 51, 836–852. 10.1016/j.visres.2010.10.017 20965208

[B22] Spering M. Schütz A. C. (2016). How eye movements improve vision and action – comment on Vickers. Curr. Issues Sport Sci. 2016, 7547–7552. 10.15203/CISS_2016.114

[B23] Spering M. Schütz A. C. Braun D. I. Gegenfurtner K. R. (2011). Keep your eyes on the ball: smooth pursuit eye movements enhance prediction of visual motion. J. Neurophysiol. 105, 1756–1767. 10.1152/jn.00344.2010 21289135

[B24] Taube J. Muller R. Ranck J. (1990). Head-direction cells recorded from the postsubiculum in freely moving rats. I. Description and quantitative analysis. J. Neurosci. 10, 420–435. 10.1523/JNEUROSCI.10-02-00420.1990 2303851 PMC6570151

[B25] Williams A. M. Singer R. N. Weigelt C. (1998). “Visual search strategy in ‘live' on- court situations in tennis: an exploratory study,” in Science and Racket Sports II, eds Lee A. Maymard I. Hughes M. Reilly T. (London: E & FN Spon), 121–218.

[B26] Yoder R. M. Taube J. S. (2009). Head direction cell activity in mice: robust directional signal depends on intact otolith organs. J. Neurosci. 29, 1061–1076. 10.1523/JNEUROSCI.1679-08.2009 19176815 PMC2768409

